# The future of network neuroscience

**DOI:** 10.1162/NETN_e_00005

**Published:** 2017-02-01

**Authors:** Olaf Sporns

**Affiliations:** Department of Psychological and Brain Sciences, Indiana University, Bloomington IN 47405 and Indiana University Network Science Institute, Indiana University, Bloomington IN 47405

## Abstract

Understanding the brain represents one of the most profound and pressing scientific challenges of the 21st century. As brain data have increased in volume and complexity, the tools and methods of network science have become indispensable for mapping and modeling brain structure and function, for bridging scales of organization, and for integrating across empirical and computational methodologies. The creation of a new journal, *Network Neuroscience*, will contribute to guiding this emerging and interdisciplinary field in new directions.

Ever since the days of Golgi and Cajal, our view of the brain has been that of a vast collection of discrete elements—an assembly of neurons that serve as the brain’s basic functional and computational units. Some of the greatest advances of neuroscience have revealed the workings of these elementary units, from the molecular structures of their channels and receptors, to their intricate morphology, the elementary processes of neurophysiology, and the links between neuronal activity and behavior.

More recently, our perspective is expanding, partly as a result of two intersecting developments. On the one hand, our experimental techniques for observing brain structure and function have dramatically increased the range, the sensitivity, and the comprehensive scope of neuroscience data. Many of these data are relational in nature—they involve the large-scale mapping and recording of anatomical and functional interactions in neuronal systems, often across multiple scales. On the other hand, the analytic methods and theoretical concepts that underpin the science of complex networks have made a significant impact in many disciplines, from the social to the biological sciences. Networks are core phenomena, whether one studies the spread of rumors or innovations, the robustness of financial markets or the Internet, or the collective dynamics of biological systems composed of proteins, cells, and species. Network science offers a common theoretical framework that guides the study of many and diverse types of networked systems.

One such system is the brain. Networks are fundamental to brain function at all levels of organization. The study of brain networks at molecular scales draws on network approaches from genomics and systems biology. At the level of cells and circuits, network studies employ a multitude of techniques, from highly resolved anatomical mapping of the connections among neurons, to large-scale recordings of dynamic circuit activity. At the level of the whole brain, networks are derived from various physiological and imaging techniques designed to observe and estimate patterns of structural and functional brain connectivity. Importantly, these levels interact—molecular-, cellular-, circuit-, and systems-level networks jointly underpin virtually all aspects of brain structure and function. Past years have seen a sharp rise in empirical and computational research directed at understanding brain networks. At the intersection of brain and network sciences, a new field has emerged—network neuroscience. One of the aims of this field is to uncover the principles that govern the architecture and dynamic function of brain networks. Achieving this aim requires a truly interdisciplinary effort. Contributions to network neuroscience frequently involve researchers with varying backgrounds and expertise—from neurobiology, physiology, development, neuroanatomy, neuroimaging, and clinical science, to informatics, data and computer science, applied mathematics, statistical physics, and engineering.

The time has come for this new interdisciplinary community to have an intellectual home—hence, the inception of this new journal, *Network Neuroscience*. Our mission is to publish innovative scientific work that significantly advances our understanding of network organization and function in the brain across all scales, from molecules and neurons to circuits and systems. We will cover both empirical and computational studies of brain networks, addressing the structure and function of networks in all systems and all species. The scope of the journal includes contributions that address developmental, evolutionary, social, and clinical/translational aspects of neurobiological networks. While the journal will mainly publish articles that report on primary research, articles that describe significant new methods, algorithms and software tools, and network datasets are also welcome. Finally, *Network Neuroscience* recognizes that review and perspective articles can play important roles in shaping an emerging field by communicating integrative overviews across the field and beyond. All of our content will undergo rigorous peer review and will be published under a Creative Commons Attribution license, providing free access worldwide. The journal strongly supports (and for some article types, requires) the sharing of data and other research materials.

In the future, I hope our journal will become more than a repository of articles in an interesting area of science. I hope that *Network Neuroscience* can take an active role in shaping the field, by helping create a new community of researchers with common interests but very different disciplinary backgrounds. In coming years, we will continually review our progress in this area and seek to incorporate new ways to facilitate interdisciplinary research, discussion, and exchange.

I am deeply grateful to the founding members of the editorial board for their enthusiasm and support, and to our publisher, MIT Press, for providing essential expertise and resources to this endeavor. I am confident that together we will make important contributions to the future of the emerging field of network neuroscience.

